# Draft Genome Sequence of *Streptococcus* sp. X13SY08, Isolated from Murray Cod (*Maccullochella peelii peelii*)

**DOI:** 10.1128/genomeA.01470-15

**Published:** 2016-01-07

**Authors:** Yang Liu, Runying Zeng, Boqi Weng, Tuyan Luo, Qin Luo, Liwen Xu

**Affiliations:** a Central Laboratory of Fujian Academy of Agricultural Sciences, Fujian, China; b State Key Laboratory Breeding Base of Marine Genetic Resource, Third Institute of Oceanography, SOA, Xiamen, China; c Institute of Agricultural Ecology of Fujian Academy of Agricultural Sciences, Fujian, China; d Key Laboratory of Fishery Ecology and Environment, Guangdong Province, Key Laboratory of South China Sea Fishery Resources Exploitation & Utilization, Ministry of Agriculture, South China Sea Fisheries Research Institute, Chinese Academy of Fishery Sciences, Guangzhou, China

## Abstract

*Streptococcus* sp. X13SY08, isolated from freshwater Murray cod ﬁsh, likely presents a novel species of *Streptococcus*. Here, we present an annotated draft genome sequence of this species, which will improve our understanding of its physiology and pathogenesis.

## GENOME ANNOUNCEMENT

The genus *Streptococcus* encompasses a broad range of Gram-positive catalase-negative chain-forming coccus-shaped organisms. Over the last decade, most novel streptococcal species have been isolated from animal sources (1, 2). Animal streptococci have been isolated from a wide range of environments (3) and some of them have been associated with a variety of diseases such as endometritis, respiratory infections, endocarditis, meningitis, arthritis, and mastitis (4). On 18 March 2015, we isolated *Streptococcus* sp. X13SY08 from the orbital tissue of a Murray cod with lesions of panophthalmitis using Columbia blood agar plates (bioMérieux). This strain has been deposited in the Marine Culture Collection of China (accession number MCCC 1A10884). Analysis of the 16S rRNA gene sequence (GenBank accession number KT223645), showed the highest similarity with *Streptococcus ovis* DSM 16829^T^ (95.8%), and physiological and biochemical features indicated that *Streptococcus* sp. X13SY08 likely represents a new species in the *Streptococcus* group, making it the first known naturally occurring strain in this clade that can infect Murray cod. This genome sequence may provide fundamental molecular information on the growth characteristics, genetic characteristics, and pathogenesis of *Streptococcus* sp. X13SY08.

The genome of *Streptococcus* sp. X13SY08 was sequenced using Illumina/Solexa MiSeq technology at the Guangzhou Gene Denovo Bio-Technology Co., Ltd. (Guangzhou, China). A library with a fragment length of 500 bp was constructed, and a total of 269 Mbp paired-end reads of 300-bp length were generated. Approximately 200 Mbp high-quality reads, which provided a 128-fold depth of coverage were assembled with SOAPdenovo version 1.05 (5). Protein-coding sequences were predicted by Glimmer software version 3.0 (6) using default parameters and annotated using BLAST searches of nonredundant protein sequences from the NCBI, Swiss-Prot and TrEMBL, COG (7), and KEGG (8) databases. For gene annotation, only significant BLAST matches with *E* values ≤10^−5^ were adopted. Ribosomal RNA genes were detected using RNAmmer software version 1.2 (9), and tRNA genes were detected using tRNAscan-SE (10).

The *Streptococcus* sp. X13SY08 genome consists of 1,548,621 bases (*N*_50_, 1,548,621 bp) in 5 contigs, with a G+C content of 40.7%. There are 1,756 putative coding sequences, 28 tRNA genes, and 3 rRNA clusters. Gene cluster that participates in the synthesis of Linaridin was detected by antiSMASH 2.0. Genus *Streptococcus* are often recognized as the causative agent of a highly contagious and fatal disease characterized by meningitis and panophthalmitis in animals (4), so we predict pathogenicity of strain X13SY08 through bioinformatics methods. Prophage identiﬁcation using the PHAST search tool (11) showed 0 prophage regions in the *Streptococcus* sp. X13SY08 genome. No known toxins were identiﬁed in the coding sequence of *Streptococcus* sp. X13SY08 by the Web server VirulenceFinder (12) or by comparing the protein sequences with the VFDB (13) and DBETH (14) toxin databases using BLASTp (15). The genome data will represent a solid platform for further characterization and exploitation of the metabolic features linked to the physiology and pathogenesis of *Streptococcus* sp. X13SY08.

### Nucleotide sequence accession numbers.

The *Streptococcus* sp. X13SY08 whole-genome shotgun (WGS) project has the project accession number LFYO00000000. The version number is LFYO02000000 and consists of sequences LFYO02000001 to LFYO02000005.
